# The lycopene β-cyclase plays a significant role in provitamin A biosynthesis in wheat endosperm

**DOI:** 10.1186/s12870-015-0514-5

**Published:** 2015-05-07

**Authors:** Jian Zeng, Cheng Wang, Xi Chen, Mingli Zang, Cuihong Yuan, Xiatian Wang, Qiong Wang, Miao Li, Xiaoyan Li, Ling Chen, Kexiu Li, Junli Chang, Yuesheng Wang, Guangxiao Yang, Guangyuan He

**Affiliations:** The Genetic Engineering International Cooperation Base of Chinese Ministry of Science and Technology, The Key Laboratory of Molecular Biophysics of Chinese Ministry of Education, College of Life Science and Technology, Huazhong University of Science and Technology, Wuhan, China

**Keywords:** Lycopene, Lycopene β-cyclase, β-carotene, Provitamin A, RNA interference, Wheat

## Abstract

**Background:**

Lycopene β-cyclase (LCYB) is a key enzyme catalyzing the biosynthesis of β-carotene, the main source of provitamin A. However, there is no documented research about this key cyclase gene’s function and relationship with β-carotene content in wheat. Therefore, the objectives of this study were to clone *TaLCYB* and characterize its function and relationship with β-carotene biosynthesis in wheat grains. We also aimed to obtain more information about the endogenous carotenoid biosynthetic pathway and thus provide experimental support for carotenoid metabolic engineering in wheat.

**Results:**

In the present study, a lycopene β-cyclase gene, designated *TaLCYB*, was cloned from the hexaploid wheat cultivar Chinese Spring. The cyclization activity of the encoded protein was demonstrated by heterologous complementation analysis. The *TaLCYB* gene was expressed differentially in different tissues of wheat. Although *TaLCYB* had a higher expression level in the later stages of grain development, the β-carotene content still showed a decreasing tendency. The expression of *TaLCYB* in leaves was dramatically induced by strong light and the β-carotene content variation corresponded with changes of *TaLCYB* expression. A post-transcriptional gene silencing strategy was used to down-regulate the expression of *TaLCYB* in transgenic wheat, resulting in a decrease in the content of β-carotene and lutein, accompanied by the accumulation of lycopene to partly compensate for the total carotenoid content. In addition, changes in *TaLCYB* expression also affected the expression of several endogenous carotenogenic genes to varying degrees.

**Conclusion:**

Our results suggest that *TaLCYB* is a genuine lycopene cyclase gene and plays a crucial role in β-carotene biosynthesis in wheat. Our attempt to silence it not only contributes to elucidating the mechanism of carotenoid accumulation in wheat but may also help in breeding wheat varieties with high provitamin A content through RNA interference (RNAi) to block specific carotenogenic genes in the wheat endosperm.

**Electronic supplementary material:**

The online version of this article (doi:10.1186/s12870-015-0514-5) contains supplementary material, which is available to authorized users.

## Background

Carotenoids are important natural isoprenoid pigments synthesized in plants that have essential roles in protecting against excess light energy and oxidative damage, and in light-harvesting [1,2]. Their provitamin A activity and antioxidant properties are their most attractive functions. β-carotene is the major and most effective vitamin A precursor among carotenoids, and plays a crucial role in human health, protecting against age-related degenerative diseases, cardiovascular disease, certain cancers and vitamin A deficiency (VAD) [3-5]. Generally, β-carotene is the most attractive target product for metabolic engineering.

**Figure 1:**
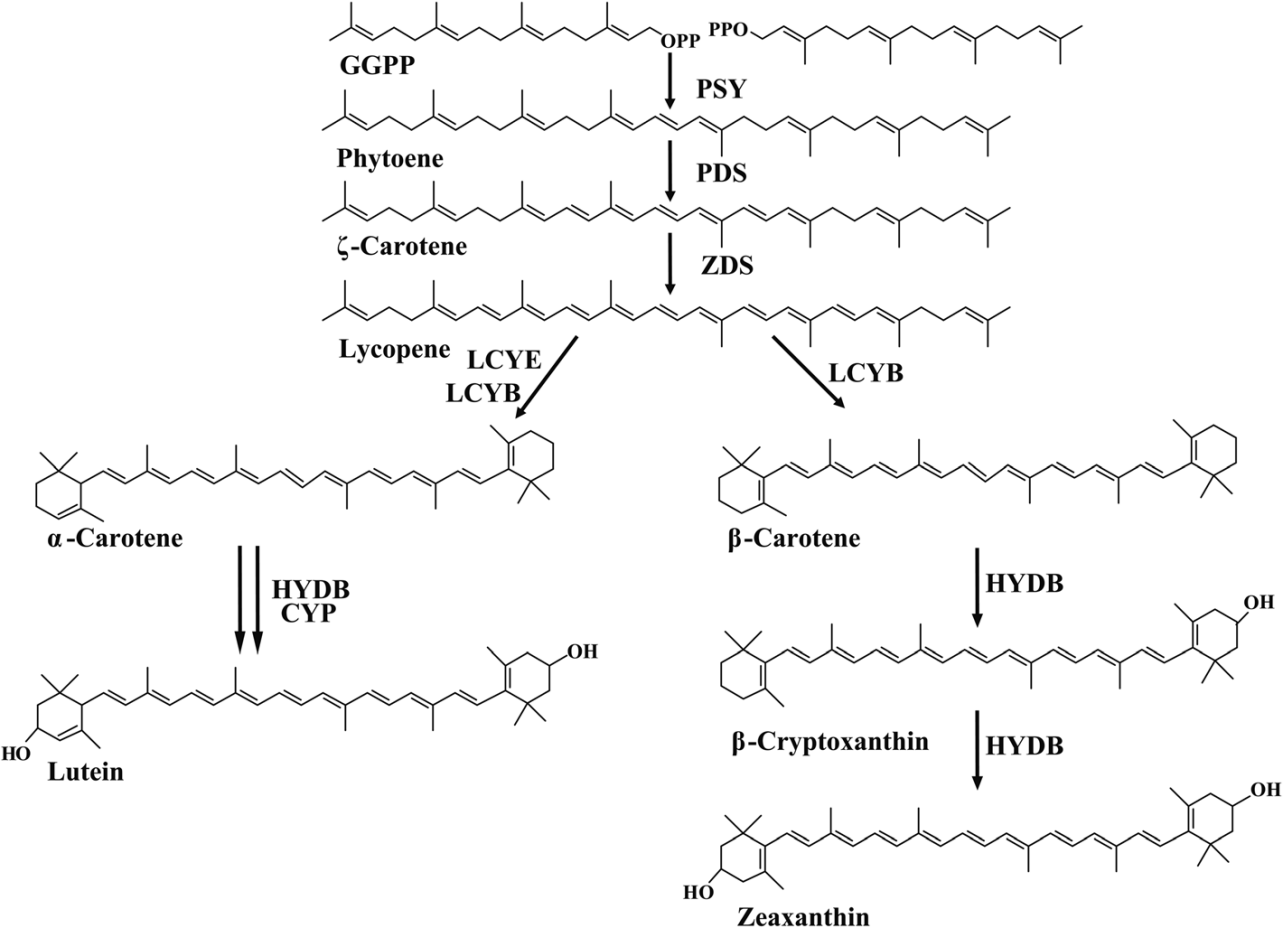
Carotenoid biosynthetic pathway in wheat. PSY, phytoene synthase; PDS, phytoene desaturase; ZDS, zeta-carotene desaturase; CRTISO, carotene isomerase; LCYB, lycopene β-cyclase; LCYE, lycopene ε-cyclase; HYDB, β-carotene hydroxylase; CYP, carotenoid ε-hydroxylase (cytochrome P450 type).

In higher plants, although the main pathway of carotenoid biosynthesis has been studied extensively [6-8], the regulatory mechanisms of carotenoid biosynthesis are still not well known. Lycopene cyclization is the first branch point of the carotenoid biosynthetic pathway, and is hypothesized to regulate the proportion of carotenes through two competing lycopene cyclases, LCYB and lycopene ε-cyclase (LCYE). In general, lycopene is cyclized by LCYE and LCYB to introduce ε and β-ionone end groups and produce α- and β-carotene, respectively (Figure 1). Only a small number of species such as *Lactuca sativa* produce ε,ε-carotene [9]. Because of the special position of lycopene cyclization, researchers have focused on the function of LCYB and its relationship with carotenoid accumulation in plants [10-13]. Plant LCYBs share similar and highly conserved functional domains, which are involved in many reactions in β-ionone catalysis [10,14].

Through the deeper understanding of the benefits of carotenoids for human health, scientists have been prompted to explore effective methods to increase the carotenoid composition and content in plants, especially in staple crops. However, precise carotenoid metabolic engineering in crop plants has been hindered by limited data about the endogenous regulation of carotenogenic genes despite recent progress in staple crops [15-17]. Thus, the first step to understanding how carotenoids are biosynthesized is to identify the related key enzymes and clone the relevant genes.

Wheat is one of the most important cereal crops in the world [18]. Given the huge daily consumption of wheat-based products in populations worldwide, increasing the β-carotene content in wheat grains could significantly impact VAD. Although carotenoids are one of the major pigments that affect the nutritional value of wheat [19], wheat grains have a very low carotenoid content and mainly accumulate lutein, which lacks provitamin A activity. To improve the carotenoid or provitamin A content in wheat, the detailed regulation of carotenoid biosynthesis must be clarified. So far, the polyploid characteristics and huge size of the wheat genome have been substantial barriers to identifying and cloning key carotenoid biosynthetic genes. Only a few carotenoid biosynthetic enzyme genes such as phytoene synthase (*PSY*) and *LCYE* have been identified [20-23]. Therefore, identifying and cloning more genes in the wheat carotenoid biosynthetic pathway will provide more information about carotenoid biosynthesis and its regulatory mechanism. According to the latest research, about 50% of the genome of hexaploid wheat has now been sequenced [24]. Although gene cloning will become easier and more precise after sequencing is completed in the future, there is still plenty of work to be done and many difficulties to be overcome. Recently, we found that endogenous *LCYB* was up-regulated by the co-expression of *CrtB* and *CrtI* in transgenic wheat, which resulted in an increase in the total carotenoid and provitamin A contents [25]. However, there is no documented research about this key cyclase gene’s function and its relationship with β-carotene content in wheat. Therefore, the objectives of this study were to clone *TaLCYB* and characterize its function and relationship with β-carotene biosynthesis in the wheat grain. We also aimed to obtain more information about the endogenous carotenoid biosynthetic pathway and thus provide experimental support for carotenoid metabolic engineering in wheat.

## Results

### Cloning and sequence analysis of *TaLCYB*

**Figure 2:**
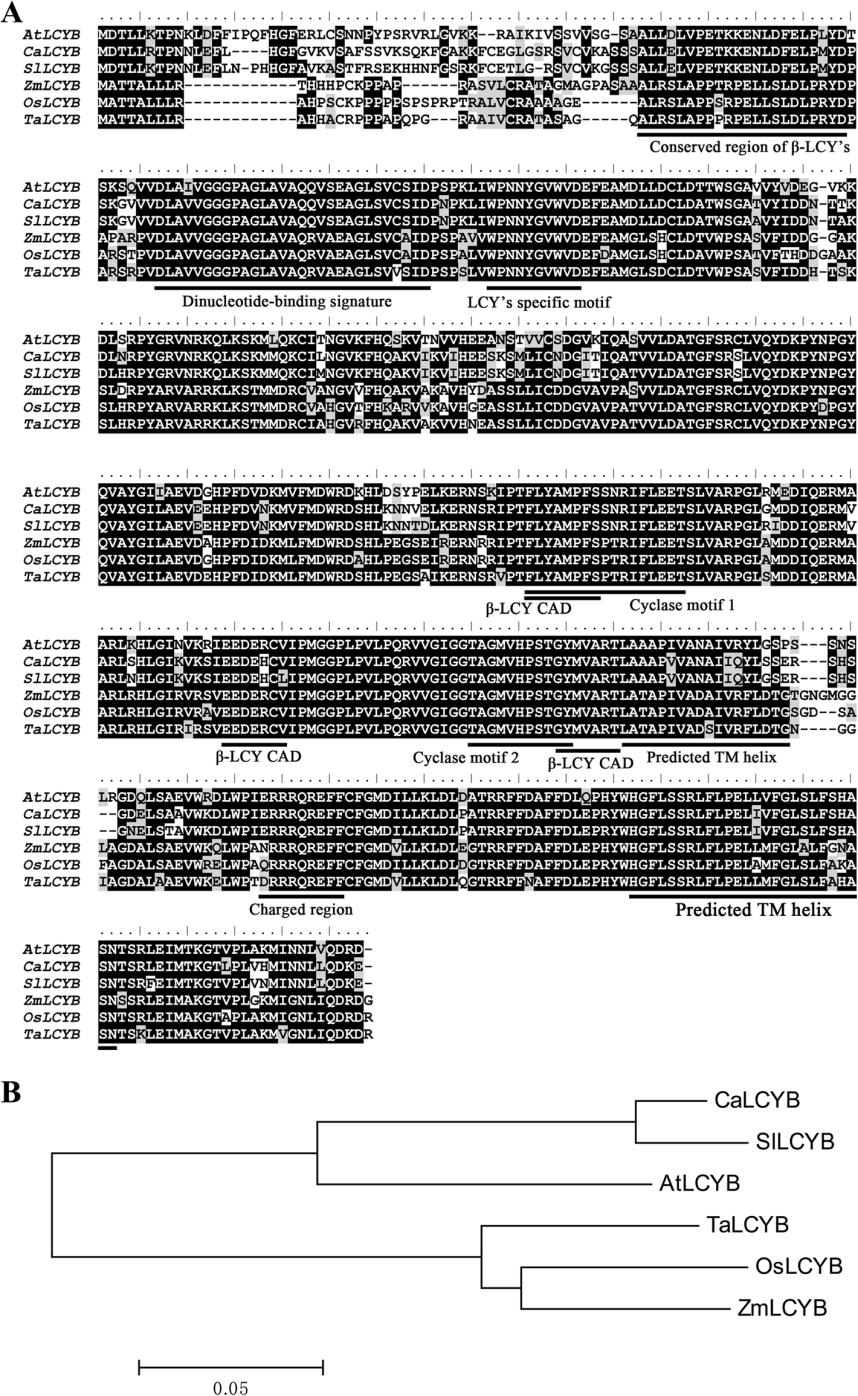
Comparative alignment and phylogenetic tree of lycopene β-cyclase. **(A)** The alignment was created using ClustalW. The amino acid residues which are identical in all sequences are shown in white text on a black background, whereas different residues are shown in black text on a white background. Characteristic regions of plant LCYBs are indicated under the LCYB sequence: Conserved LCYB region, Di-nucleotide binding site, LCY’s specific motif, Cyclase motifs (CM) I and II, Predicted TM helices, Charged region and β-LCY CAD (Catalytic Activity Domain). AtLCYB: *A. thaliana* lycopene β-cyclase; CaLCYB: *C. annuum* lycopene β-cyclase; SlLCYB: *S. lycopersicum* lycopene β-cyclase; ZmLCYB: *Z. mays* lycopene β-cyclase; OsLCYB: *O. sativa* lycopene β-cyclase. **(B)** The multiple alignments were generated by ClustalW and the phylogenetic tree was constructed with MEGA4.0 using a bootstrap test of phylogeny with minimum evolution test and default parameters.

A 1,455 bp full-length cDNA of *LCYB* from common wheat was isolated through an *in silico* cloning strategy. The full-length cDNA of the *LCYB* gene was designated *TaLCYB* (GenBank Accession No.: JN622196.1). Comparison of the obtained cDNA sequence with the gDNA sequences of wheat revealed an intronless structure. Based on the latest database of the International Wheat Genome Sequencing Consortium, *TaLCYB* was localized on 6AS and 6DS (https://urgi.versailles.inra.fr/blast/blast.php). The ORF encoded a polypeptide of 484 amino acid residues with a predicted relative molecular mass of 53.3 kDa containing a predicted plastid transit peptide of 30 amino acids. Multiple alignment showed that TaLCYB shared a significant degree of sequence identify with other LCYB proteins in monocots (86.3% sequence identity with OsLCYB from *O. sativa*, 86.2% with ZmLCYB from *Z. mays*), and relatively lower homology with LCYB proteins from dicot species, such as *C. annuum*, *A. thaliana*, *S. lycopersicum* (67.4%, 67.1% and 66.5% respectively). Conserved motifs analysis (Figure 2A) showed a conserved β-LCY region, a dinucleotide-binding signature, a LCY-specific motif, cyclase motifs I and II, a charged region, two predicted TM helices and three β-LCY CAD regions (Catalytic Activity Domain). These domains were shown to be essential for LCYB activity [10,26,27]. A phylogenetic tree was constructed based on the amino acid sequence alignment of TaLCYB and five other plant LCYBs from GenBank (Figure 2B). These results suggested that TaLCYB isolated from wheat was a genuine member of the plant lycopene β-cyclase family.

### Functional characterization of TaLCYB in *E. coli*

**Figure 3:**
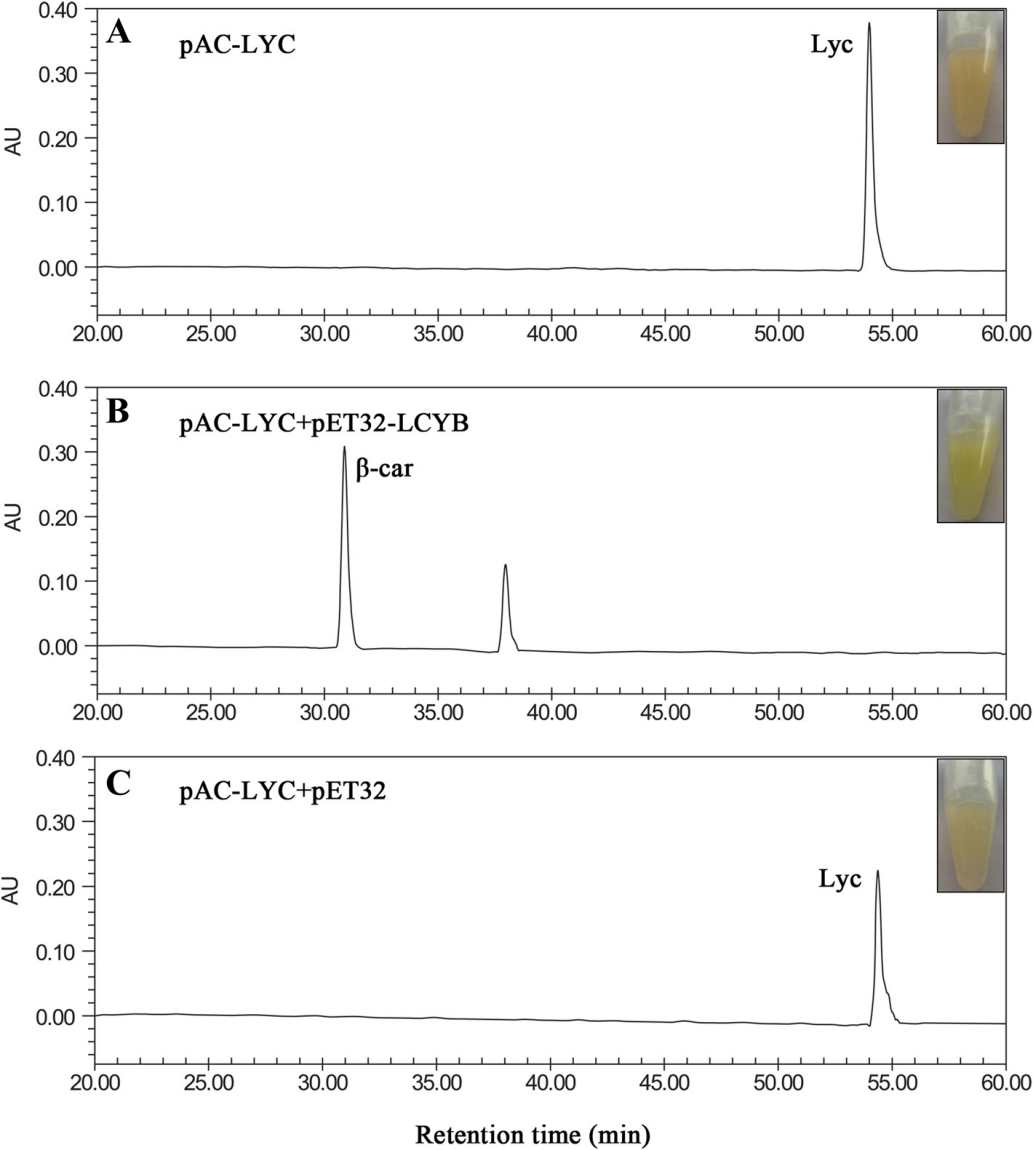
Reverse phase HPLC analysis of carotenoids accumulated in *E. coli* BL21 strain complemented with *TaLCYB*. Carotenoids were extracted from suspension cultures of cells with different plasmids **(A)** plasmids pAC-LYC; **(B)** pAC-LYC + pET32-LCYB; **(C)** pAC-LYC + pET32.

While the LCYB conserved motifs gave an indication of the enzymatic function of the encoded protein, they could not fully determine or reflect its cyclic function *in vivo*. To investigate the function of TaLCYB, an *in vivo* analysis using *E. coli* BL21 was conducted. *TaLCYB* was cloned into pET32α(+), which was then designated pET32-LCYB. *E. coli* strain BL21 was co-transformed with pAC-LYC, which contains genes for lycopene biosynthesis, and pET32-LCYB. Carotenoids were extracted from the bacterial cells and analyzed by High Performance Liquid Chromatography (HPLC). As shown in Figure 3, HPLC analysis of BL21 extracts showed that the strains containing pAC-LYC or pAC-LYC + pET32α(+) exhibited a single peak, whose retention time and absorbance spectrum corresponded to lycopene, and the cultures appeared pink. In contrast, extracts from pAC-LYC + pET32-LCYB cells mainly accumulated β-carotene, the cultures turned yellow with an undefined peak (maybe an isomer of β-carotene), and lycopene was virtually undetectable. These results demonstrated that TaLCYB was a functional β-cyclase in the carotenoid biosynthetic pathway in *E. coli*, which could convert lycopene to β-carotene.

### *TaLCYB* is expressed in different tissues including developing grains of common wheat

**Figure 4:**
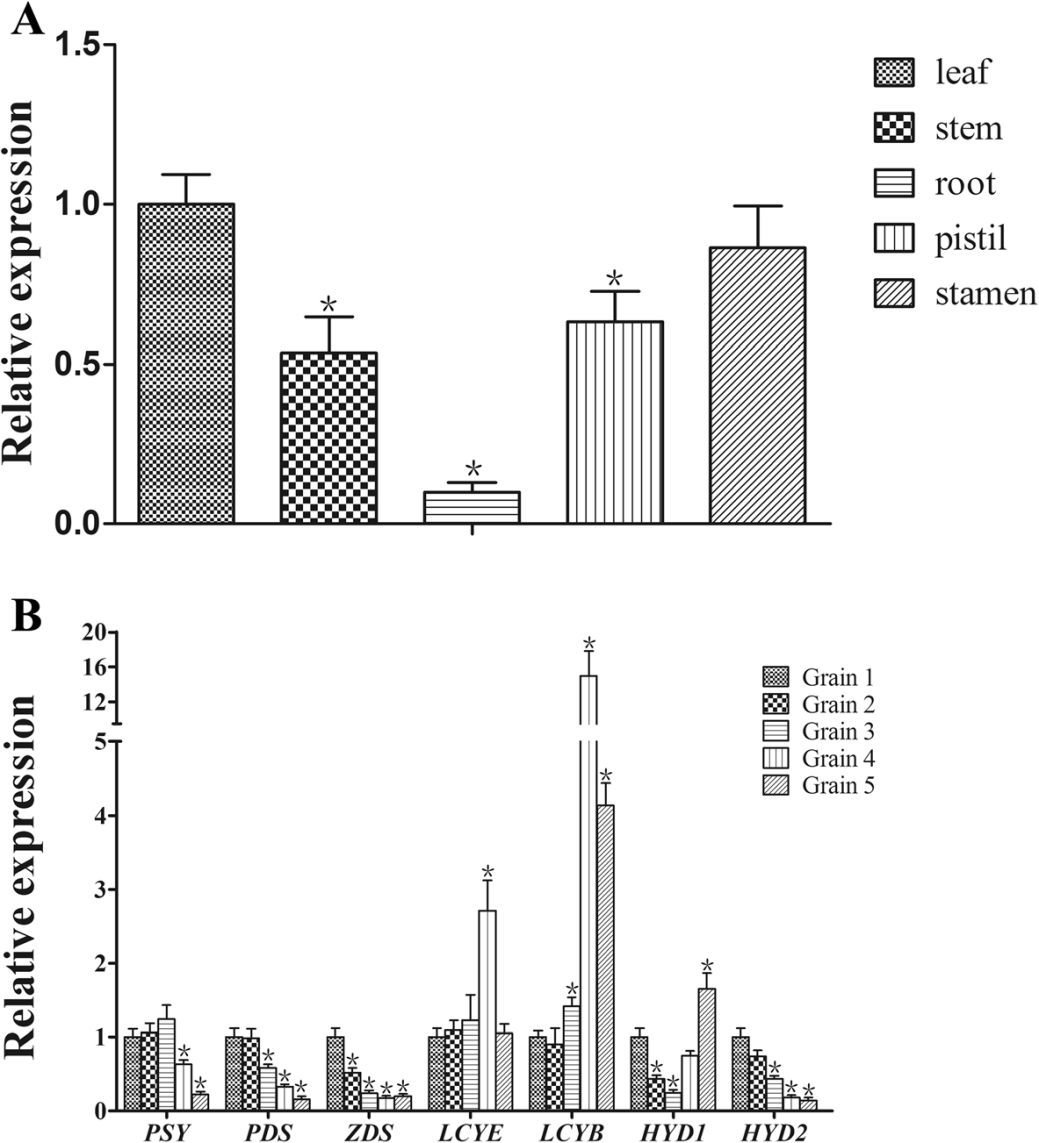
Expression patterns of wheat *TaLCYB* revealed by qRT-PCR analysis. **(A)** Expression patterns of *TaLCYB* in different tissues. **(B)** Expression levels of the endogenous carotenoid biosynthetic genes in developing grains of common wheat. Poly(A)^+^ mRNA of 200 ng was subjected to reverse transcription, and served as the qPCR template. qPCR results for each gene were performed in three biological replicates with three technical repeats each and all data are shown as Mean ± SEM. Single asterisk indicates significant differences in the expression levels between controls at *P* = 0.05 probability level.

To assess the spatial and temporal expression patterns of *TaLCYB* in different wheat tissues, quantitative PCR (qPCR) was carried out with RNA extracted from leaves, stems, roots, pistils, stamens and five developmental stages of grains: Grain 1 (4–10 days after pollination (DAP)), Grain 2 (10–16 DAP), Grain 3 (16–20 DAP), Grain 4 (20–25 DAP) and Grain 5 (25–35 DAP). As shown in Figure 4, *TaLCYB* was expressed in all of these tissues. The highest expression of the *TaLCYB* gene was observed in the leaf followed by the stamen, pistil, stem and root. In developing grains, it was interesting that the expression of *TaLCYB* always remained at a relatively high level, particularly at the later stages. *TaLCYB* expression peaked (15-fold) at 20–25 DAP (Grain 4) in common wheat and then decreased, but still remained at 4.1-fold in Grain 5 when compared with Grain 1 (Figure 4B).

### Common wheat carotenoid accumulation presents a decreasing tendency in developing grains

The carotenoid composition of wheat grains at different developmental stages was determined by HPLC analysis. As shown in Additional file 1: Figure S1, detailed HPLC analysis revealed a decreasing tendency in total carotenoid content during grain development. The β-carotene content in the wheat grains also showed a decreasing tendency, despite *TaLCYB* expression remaining at a high level during later developmental stages. Carotenoid pigments, encompassing lutein, zeaxanthin, β-cryptoxanthin, α-carotene and β-carotene, were measured and their concentrations all decreased progressively during grain development. At the last stage, the main carotenoids were lutein, zeaxanthin and β-carotene (Table 1).

**Table 1:** **Cartenoids content and compositions in developing grains**

**Grain**	**Lutein**	**Zeaxanthin**	**β-cryptoxanthin**	**α-carotene**	**β-carotene**	**Total carotenoid**
	**(μ** **g g** ^**−1**^ **)**	**(** **μg g** ^**−1**^ **)**	**(** **μg g** ^**−1**^ **)**	**(** **μg g** ^**−1**^ **)**	**(** **μg g** ^**−1**^ **)**	**(** **μg g** ^**−1**^ **)**
1	5.52 ± 0.61a (60%)	0.60 ± 0.09a (7%)	0.27 ± 0.03a (3%)	0.36 ± 0.05a (4%)	2.35 ± 0.31a (26%)	9.10 ± 1.82a
2	4.70 ± 0.42b (59%)	0.56 ± 0.08a (7%)	0.23 ± 0.02a (3%)	0.33 ± 0.04a (4%)	2.12 ± 0.29b (27%)	7.94 ± 0.95b
3	3.80 ± 0.88c (62%)	0.40 ± 0.06b (6%)	0.17 ± 0.02b (3%)	0.32 ± 0.04a (5%)	1.47 ± 0.21c (24%)	6.16 ± 0.80c
4	1.93 ± 0.29d (50%)	0.22 ± 0.03c (6%)	0.08 ± 0.01c (2%)	0.31 ± 0.03a (8%)	1.32 ± 0.15d (34%)	3.86 ± 0.54d
5	0.52 ± 0.08e (53%)	0.17 ± 0.03c (17%)	ND	ND	0.29 ± 0.03e (30%)	0.98 ± 0.16e

Data represent the average carotenoid content (±SEM) of grains from five individual ears per line. Different letters indicate significant differences (*P* = 0.05) in carotenoid pigment content were determined by Tukey’s HSD test. Values in parentheses represent the percentages of each carotenoid composition relative to the total content. ND = not detected.

In parallel with the carotenoid content analysis, the expression of carotenogenic genes in developing wheat grains was also analyzed. As shown in Figure 4B, *TaPSY*, *TaPDS*, *TaZDS* and *TaHYD2* showed similar expression patterns in developing grains, presenting a declining tendency. The expression pattern of *TaLCYE* was similar to that of *TaLCYB*; both maintained a relatively high expression level in all development stages (Grains 1–5). *TaHYD1* expression showed a decline in Grains 1–3, but was up-regulated in Grains 4–5. In the last two stages, carotenogenic gene expression was dramatically reduced in comparison with the early stages, except that *TaLCYE* and *TaLCYB* were slightly down-regulated and *TaHYD1* was up-regulated. Overall, the carotenogenic genes had relatively stable expression levels in the early stages (Grains 1–3). This suggested that carotenoids were synthesized at a stable rate during the early stages.

### Expression patterns of *TaLCYB* under different abiotic stresses and their effects on β-carotene accumulation

qPCR was performed to analyze the expression level of *TaLCYB* under different abiotic stresses, such as strong light, darkness and cold. As shown in Additional file 1: Figure S2, *TaLCYB* transcripts were up-regulated by strong light and cold, and inhibited by darkness. Under strong light conditions, dramatic and fast changes in *TaLCYB* expression were observed. The expression of *TaLCYB* reached a peak (about 8.5-fold) after 4 h under strong light treatment. By contrast, changes in *TaLCYB* expression were more gradual under cold, with the highest expression (1.4-fold) at 8 h after treatment. As the expression of *TaLCYB* was dramatically induced by strong light, the carotenoid profiles of leaves at different treatment times were analyzed by HPLC (Additional file 1: Figure S3). Notably, the β-carotene content variation seemed concurrent with changes of *TaLCYB* expression; the highest expression level corresponded to the maximum β-carotene content (Additional file 1: Figure S2B). The expression of other upstream genes in the pathway such as *TaPSY*, *TaPDS*, *TaZDS* and *TaLCYE* was also up-regulated by strong light (Additional file 1: Figure S4). These results suggested a correlation between *TaLCYB* expression and β-carotene content in wheat.

### *TaLCYB* RNAi increases lycopene by decreasing β-carotene accumulation in the seeds

**Figure 5:**
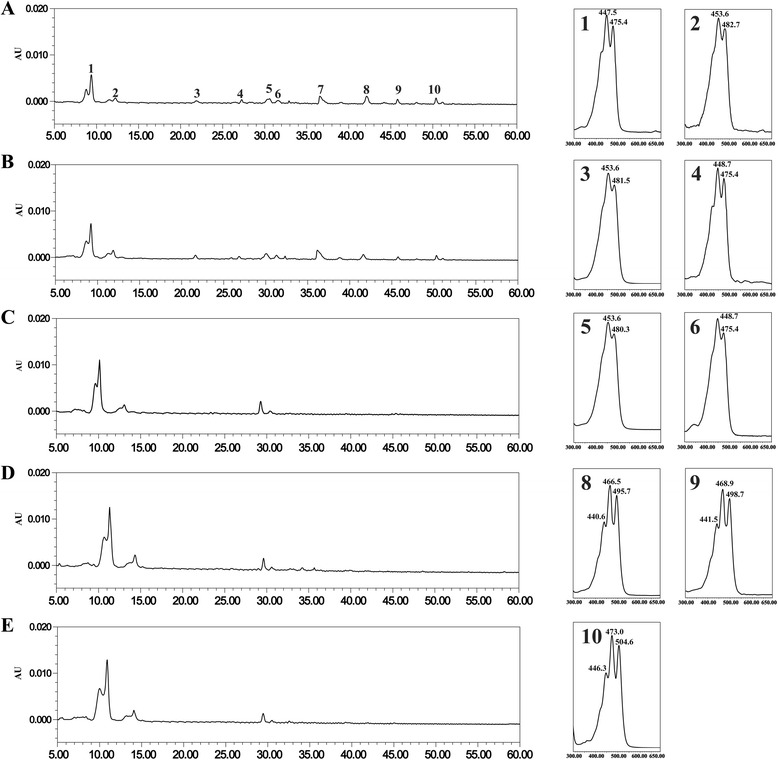
HPLC characterization of carotenoids extracted from grains of T_3_ transgenic and control wheat. **(A)** BI-9; **(B)** BI-6; **(C)** BI-2; **(D)** VC-10 (transgenic vector control); **(E)** Chinese Spring (wild-type); Peak 1, lutein; Peak 2, zeaxanthin; Peak 3, β-cryptoxanthin; Peak 4, α-carotene; Peak 5, *trans*-β-carotene; Peak 6, 9-*cis*-β-carotene; Peak 7, undefined carotene; Peak 8,9, *cis*-lycopene; Peak 10, *trans*-lycopene.

To explore the function of *TaLCYB* in the wheat carotenoid biosynthetic pathway, an RNAi vector was constructed and transformed into wheat (cv. Chinese Spring). After herbicide-selective regeneration, positive transgenic wheat lines were screened out in the T_0_ generation by specific PCR-amplification of both the *bar* gene sequence and vector (sense-intron) sequence. Three transgenic lines and several lines only transformed with the pAHC25 plasmid were obtained; the latter lines were regarded as vector control lines (VC). Self-pollination of the PCR-positive transgenic plants in subsequent generations led to the identification of non-segregant RNAi transgenic lines. HPLC analysis of carotenoids showed no distinction in the carotenoid composition between the VC and wild-type. The carotenoid profiles and total carotenoid content of the transgenic lines differed from the wild-type. However, transgenic line BI-2 did not show any changes in carotenoid content or profile compared with the wild-type. Several novel carotenoids were observed in the transgenic wheat lines including lycopene, β-cryptoxanthin and α-carotene (Additional file 1: Table S1). To further analyze the carotenoid profiles, detailed HPLC analysis was carried out on the T_3_ generation, which showed significant differences in carotenoid content and composition in seeds between transgenic and control lines, implying profound changes in the carotenoid biosynthetic pathways of the transgenic lines (Figure 5). The total carotenoid content slightly decreased to 0.84 μg g^−1^ seed dry weight in BI-6 and 0.75 μg g^−1^ seed dry weight in BI-9 compared with the wild-type (0.96 μg g^−1^). In these two lines, consistent with the hypothesized silencing of *TaLCYB* genes, the β-carotene content decreased to 0.16 μg g^−1^ and 0.09 μg g^−1^ compared with the wild-type (0.22 μg g^−1^). Lycopene is the immediate precursor of lycopene β-cyclase, and was accumulated to 0.22 μg g^−1^ and 0.39 μg g^−1^ in BI-6 and BI-9, respectively. Because LCYB participates in the biosynthesis of lutein, the lutein content was also decreased to 0.22 μg g^−1^ and 0.18 μg g^−1^ in BI-6 and BI-9, respectively, compared with 0.59 μg g^−1^ in the wild-type (Table 2).

**Table 2:** **Cartenoids content and compositions in grains from transgenic wheat**

	**Lutein**	**Zeaxanthin**	**Lycopene**	**α-carotene**	**β-carotene**	**β-cryptoxanthin**
	**(** **μg g** ^**−1**^ **)**	**(** **μg g** ^**−1**^ **)**	**(** **μg g** ^**−1**^ **)**	**(** **μg g** ^**−1**^ **)**	**(** **μg g** ^**−1**^ **)**	**(** **μg g** ^**−1**^ **)**
Bobwhite	0.59 ± 0.07a	0.15 ± 0.017a	ND	ND	0.22 ± 0.025a	ND
VC	0.57 ± 0.08a	0.17 ± 0.015a	ND	ND	0.24 ± 0.03a	ND
BI-2-9	0.51 ± 0.06a	0.18 ± 0.02a	ND	ND	0.26 ± 0.03a	ND
BI-6-1	0.22 ± 0.02b	0.13 ± 0.018a	0.22 ± 0.028b	0.06 ± 0.016a	0.16 ± 0.017b	0.04 ± 0.015
BI-9-5	0.18 ± 0.015c	0.05 ± 0.014b	0.39 ± 0.06a	0.05 ± 0.016a	0.09 ± 0.022c	ND

Carotenoid composition in wheat grains from transgenic and control lines in T_3_ generation. Average of each carotenoid species are determined from five individual plants ears per line. Data represent the average carotenoid content (±SEM) of grains from five individual ears per line. Different letters indicate significant differences (*P* = 0.05) in carotenoid pigment content were determined by Tukey’s HSD test. ND = not detected.

### Decreased β-carotene content in transgenic wheat was due to down-regulation of *TaLCYB*

**Figure 6:**
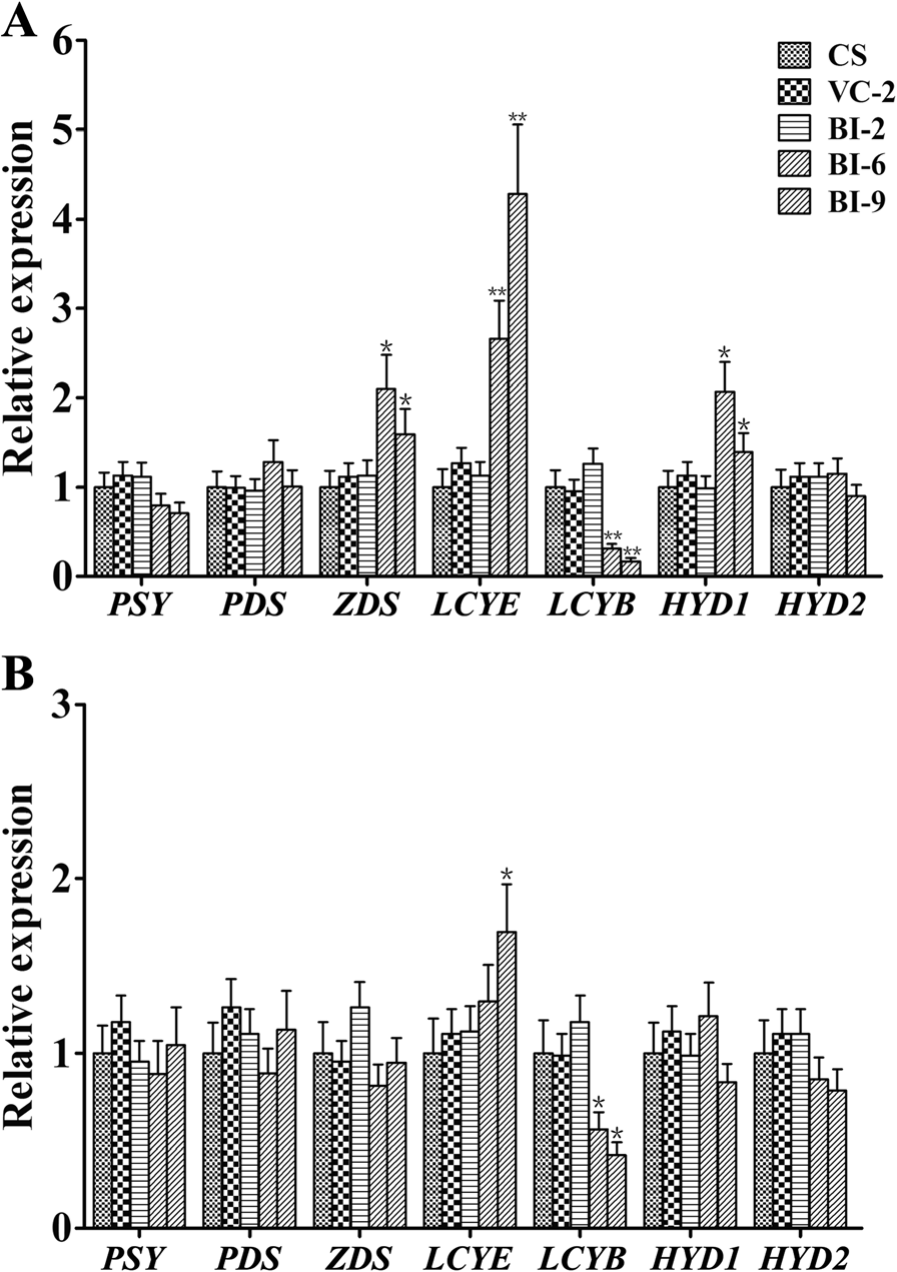
Expression levels of the endogenous carotenoid biosynthetic genes in endosperms and leaf from transgenic and control wheat lines. Gene expression levels were measured by qPCR and are determined relative to the transcript levels of the constitutively expressed β-actin gene in the same samples. Expression levels of these genes for the transformed lines are given as expression levels relative to the values for non-transformed control line Chinese Spring (CS). qPCR results for each gene were performed in three biological replicates with three technical repeats each and all data are shown as Mean ± SEM. Single asterisk and double asterisk indicate significant differences in the expression levels between control CS and transgenic lines at *P =* 0.05 or *P =* 0.01 probability level, respectively.

Transcriptional regulation of carotenogenic genes is a crucial regulatory mechanism of carotenoid accumulation in plants. A post-transcriptional gene silencing strategy was used to verify the function of *TaLCYB*. The β-carotene and lutein contents were demonstrated to be reduced through HPLC analysis. The expression levels of the endogenous carotenogenic genes were thus analyzed in both endosperms and leaves from transgenic and control lines to investigate whether the decrease of β-carotene and lutein content in transgenic lines was related with carotenogenic gene expression. As shown in Figure 6, transgenic line BI-2, VC and the wild-type showed similar expression levels for all carotenogenic genes in the endosperm. In transgenic lines BI-6 and BI-9, the expression of *TaLCYB* showed a 70% and 84% reduction, respectively. This was consistent with the HPLC results that lower expression of *TaLCYB* accompanied decreased β-carotene content. In these transgenic lines, the expression levels of *TaZDS*, *TaLCYE* and *TaHYD1* were up-regulated. The expression of *TaPSY* showed slight suppression, which was possibly correlated with the decrease in total carotenoids. The remaining carotenogenic genes such as *TaPDS* and *TaHYD2* appeared to be unaffected by the reduced *TaLCYB* expression.

Endogenous carotenogenic genes from the transgenic lines were much less affected in leaves than in the endosperm. In transgenic lines BI-6 and BI-9, the expression of *TaLCYB* was down-regulated, the other cyclase *TaLCYE* was up-regulated, and *TaHYD2* was slightly down-regulated. In the leaves of transgenic line BI-2, the expression of all carotenogenic genes showed the same transcriptional levels as the VC line and wild-type (Figure 6B). The carotenoid composition and content in the leaves of transgenic and control lines were also analyzed by HPLC; there was no distinction between the transgenic lines and control lines (data not shown).

## Discussion

Because of the nutritional value and health benefits of carotenoids, there have been many attempts to improve the carotenoid content in staple crops by metabolic engineering, especially the β-carotene content. For example, the transgenic cereal ‘Golden Rice 2’ was developed because of the deficiency of β-carotene in rice grains [28], in which the endogenous LCYB plays a crucial role in β-carotene biosynthesis. However, owing to the complexity of the wheat genome, there are limited reports on carotenoid metabolic engineering to improve the carotenoid content in wheat. One of the major limitations to metabolic engineering in wheat is the lack of a fully elucidated carotenoid biosynthetic pathway [25,28-30]. Additionally, only a few related genes have been cloned and characterized in wheat because of its complicated and huge genome, which seriously hinders the understanding of carotenoid biosynthesis in wheat. Therefore, cloning and analyzing carotenoid biosynthetic genes in wheat is very important to elucidate the carotenoid biosynthesis pathway and to improve its nutritional value by metabolic engineering. In this study, a novel wheat gene, *TaLCYB*, was identified and characterized to function as a lycopene β-cyclase. Its relationship to carotenoid biosynthesis was also investigated, in particular to β-carotene biosynthesis.

### TaLCYB has β-lycopene cyclase function according to bioinformatics analysis and heterologous complementation in *E. coli*

To provide more information about *TaLCYB*, the TaLCYB protein was analyzed by comparing its amino acid sequence with other LCYBs from monocot (OsLCYB and ZmLCYB) and dicot species (CaLCYB, AtLCYB and SlLCYB) [11,31-33]. The amino acid sequence analysis revealed that TaLCYB contains all the conserved domains of plant LCYBs (Figure 2A). For instance, β-LCY CAD regions, which have been reported as crucial to LCYB catalytic activity, were found in TaLCYB. The “Conserved region β-LCY” was also found in TaLCYB, which is regarded as a crucial factor for the association of LCYB with membrane components and for its catalytic activity. These conserved motifs showed a high degree of conservation in amino acids between monocot and dicot species (Figure 2B) [10,26,27]. However, the sequence analysis only gave a preliminary indication of the function of TaLCYB. Therefore, a heterologous complementation system was used to verify its function *in vivo*. This method has been proved to be efficient for functional characterization of carotenoid biosynthetic genes [8]. Consistent with the results of sequence analysis, TaLCYB was demonstrated to be a functional β-cyclase enzyme *in vivo*, converting lycopene to β-carotene (Figure 3).

### The endogenous expression level of *TaLCYB* is not positively correlated with β-carotene accumulation in developing grains

The tissue specificity of gene expression usually mirrors the function of the corresponding gene products in plant development. Maximal expression in leaves should be associated with the photosynthetic system and photo-protective function where carotenoids are a key component [34,35]. In developing grains, it was found that *TaLCYB* reached its highest expression at 20–25 DAP (Grain 4), suggesting that at later developmental stages grains may also have high ability to synthesize β-carotene compared with the early stages. The decreasing tendency of β-carotene content meant that increased β-ring cyclization capacity did not present as a large amount of β-carotene accumulation in developing wheat grains (from 2.35 μg g^−1^ to 0.29 μg g^−1^). This phenomenon might be explained by the β-carotene produced by increased synthesis being rapidly transformed into downstream compounds, resulting in a net decrease in β-carotene content. The total carotenoid content in developing wheat grains also showed a decreasing trend, which was the same as in Qin *et al*. [20]. The high expression of *TaHYD1* in later stages also suggested high downstream synthetic capacity to some degree, which is more preferred to the β,β-branch [25]. In addition, the accumulation of carotenoids is inversely determined by the rate of carotenoid turnover, in which the activities of various carotenoid cleavage dioxygenases (*CCDs*) play a crucial role [36]. The CCD family catabolizes the turnover of different carotenoids to apo-carotenoids in various crops, such as rice and maize [37]. Experimental evidence from the expression of carotenoid cleavage dioxygenase 1 and the carotenoid content in maize endosperm demonstrates that high expression of *CCD1* accompanies lower carotenoid accumulation [37,38]. LCYB is supposed to be a key step for β,ε- and β,β-branch biosynthesis [10]. In kiwifruit and papaya, the major carotenoid was controlled by the expression level of *LCYB* [12,13]. In transgenic wheat with introduced *CrtB* and *CrtI*, constant lutein content in mature grains was still maintained despite high expression of *TaLCYB*. The relatively higher expression of *TaLCYB* and *TaLCYE* in developing grains did not translate into accumulation of the corresponding carotenoids. All of these phenomena suggest that coordination between *TaLCYB* and *TaLCYE* expression would regulate carbon flux through different branches in the wheat carotenoid pathway. They also show that there is a mechanism resulting in a net decrease due to more carotenoid compounds entering into turnover relative to the biosynthetic capacity.

### *TaLCYB* plays a crucial role in the β-carotene biosynthesis of wheat

*LCYB* and *LCYE* determine the flux towards the β,β- and β,ε-carotenoid branches. Thus, a strategy of modulating the levels of these two competing cyclases should enable the control of carotenoid composition [39]. Our results showed that the *TaLCYB* transcript could be regulated by silencing, which resulted in decreased β-carotene content and lycopene accumulation. In the transgenic lines BI-6 and BI-9, the β-carotene content showed obvious reduction (Table 2). The most dramatic down-regulation of the endogenous *TaLCYB* gene was coincident with the lowest metabolic flux into the β,β-carotene branch in BI-9. The same results were also observed in transgenic carrot with *DcLCYB1* silencing, while the β-carotene content was increased by over-expression of *DcLCYB1* [40]. Under strong light treatment, qPCR and HPLC analysis results showed that the expression level of *TaLCYB* and the β-carotene content in leaves presented the same change tendency. The increased biosynthesis of β-carotene was due mainly to the combined effects of *TaLCYB* and *TaPSY* up-regulation. In the present study, lycopene and α-carotene were the carotenoid composition in the silenced transgenic lines, but these were undetectable in the VC and wild-type. Lycopene and α-carotene are direct substrates or products of LCYB, which indicates that the silencing of *TaLCYB* simultaneously affects its upstream and downstream products. TaLCYB is also required for lutein synthesis; thus, one of the possible reasons for the decreased lutein content could be that TaLCYB is the key enzyme of lutein biosynthesis in wheat seeds. Additionally, it is also possible that changing the pigment composition could regulate the expression of enzymes. For example, the expression of *TaHYD1*, which is more related with β,β-branch synthesis, was up-regulated in the transgenic lines while the expression of *TaHYD2*, which is more related with β,ε-branch synthesis, maintained stable expression. In addition, cyclases and hydroxylases are thought to form a protein complex to function; thus, the down-regulation of *TaLCYB* may impair protein complex formation and influence the biosynthesis of lutein or other carotenoids [41]. The accumulation of lycopene partly compensated for the decrease of β-carotene and lutein in the total carotenoid content, and also showed that carotenoid flux was a whole, opening the possibility for the metabolic engineering of compounds in the carotenoid pathway through an appropriate strategy to modulate the expression of carotenogenic genes in the carotenoid biosynthetic pathway. Generally, all these results suggested that TaLCYB acts as the key enzyme in the downstream carotenoid biosynthetic pathway and determines the β-carotene synthesis capacity.

### Down-regulation of *TaLCYB* transcripts affects the expression of other genes in the carotenoid biosynthetic pathway of wheat

The expression levels of related endogenous carotenogenic genes are often altered when introducing exogenous genes, and simultaneously alter the levels of carotenoids in the biosynthetic pathway. This phenomenon has been documented in tomato leaves, potato tubers and maize kernels [29,30,42]. In the present study, the expression of endogenous carotenogenic genes was analyzed in endosperms and leaves from transgenic and control lines. In endosperms, the expression level of *TaLCYB* was reduced, *TaHYD1* and *TaLCYE* were up-regulated, *TaHYD2* maintained stable expression, and *TaPSY* was slightly down-regulated (Figure 6A). In previous research, *TaHYD1* was shown to be more related with β,β-branch synthesis, while *TaHYD2* was more related to β,ε-branch synthesis. This implies that the down-regulation of *TaLCYB* led to more flux to the β,ε-branch, accompanied by the occurrence of α-carotene and the accumulation of lycopene. Other intermediates such as phytoene, phytofluene or ζ-carotene upstream of the carotenoid pathway were not detected, which was probably due mainly to the expression of *TaZDS* and *TaPDS* without significantly altering compared with the wild-type. Because of insufficient TaLCYB, the expression of *TaHYD2* in the transgenic lines was stable. Although *TaHYD1* and *TaLCYE* were up-regulated, the repression of *TaLCYB* could explain the reduction of zeaxanthin and lutein. Since ubiquitous feedback or forward regulation exists in the carotenoid biosynthetic pathway and *PSY* has frequently been reported as the rate-limiting gene in non-green plant tissues [28,43-45], the accumulation of lycopene may lead to feedback regulation to suppress *TaPSY* accompanied by a decrease in total carotenoid content, which might suggest an attenuated phytoene synthesis capacity as a consequence of *TaLCYB* down-regulation. These results are consistent with a previous report on the endosperm of transgenic lines that were transformed with the exogenous genes *CRTB* or/and *CRTI* [25]. However, in the leaves of our transgenic lines (Figure 6B), carotenogenic gene transcripts and carotenoid content and composition did not show dramatic changes. There are four possible explanations for this phenomenon. (1) It is possible that the silencing efficiency was too low or that hexaploid wheat has other *LCYB* gene copies that might not be totally silenced by *LCYB* RNAi, and which might have compensated for β-ring formation. For example, there are two types of LCYBs expressed differently in the diploid species tomato; LCYB1 is active in green tissues, while LCYB2/CYCB functions only in chromoplast-containing tissues such as ripening fruit [46]. The same phenomenon has been observed with the other wheat carotenoid biosynthetic genes, such as the different expression patterns of *TaHYD1* and *TaHYD2* in vegetative tissues and developing grains of wheat. Additionally, our results suggest that *TaLCYB* may be located on 6AS and 6DS according to the latest sequencing of hexaploid wheat [24]; these results indicate that *TaLCYB* might have other copies in hexaploid wheat. (2) The regulatory mechanism of carotenoid biosynthesis in leaves is more stringent to prevent disruption of photosynthesis, because carotenoids are an important part of the photosynthetic apparatus [35,47]. (3) The enzyme activity of LCYB is sufficient for product synthesis to maintain normal photosynthesis in leaves, so incomplete silencing might not affect carotenoid biosynthesis in leaves. (4) The effects in leaves are very weak, which correlates with the low reduction in co-suppressed *TaLCYB*. Thus, it also appears likely that the lines with stronger co-suppression of *TaLCYB* than those we obtained were not viable as a critical level of photosynthetically relevant carotenoids could not be attained.

## Conclusions

In summary, this study demonstrates that *TaLCYB* is a genuine carotenoid biosynthetic gene. The silencing of *TaLCYB* led to a decrease of β-carotene content and altered the carotenoid profile and accumulation, accompanied by changes in the expression of endogenous carotenogenic genes to varying degrees. This provides new ideas and means for improving the total carotenoid content or specific carotenoid products by metabolic engineering in wheat. For example, the combination of RNAi-induced gene silencing with overexpression of upstream synthetic genes constitutes a strategy to improve specific carotenoid products in wheat. Generally, our data demonstrate that LCYB is a key enzyme of β-carotene biosynthesis and plays an essential role in the regulation of provitamin A biosynthesis in wheat, controlling flux to the downstream carotenogenic pathway. Although the precise regulatory mechanisms of carotenoid biosynthesis in wheat need to be investigated in future, these findings increase our knowledge of carotenoid biosynthesis in wheat and provide novel implications for wheat carotenoid bioengineering.

## Methods

### Plant materials and treatments

Wheat (*Triticum aestivum* L. cv. Chinese Spring) plants were grown in the experimental field of Huazhong University of Science and Technology in Wuhan, China. Developing grains were collected between 5 and 35 DAP at 5-day intervals. Leave, stem, root, stamen and pistil tissues were collected from wheat plants in the field. Abiotic stresses including cold, darkness and strong light (800 μmol · m^−2^ · s^−1^) were used to examine their effect on the expression of *TaLCYB*. The 10-day-old seedlings were transferred respectively into a dark box, cold room (4°C) or in a growth chamber at constant temperature of 25°C as a control. Strong light stress was imposed by increasing light intensity to 800 μmol · m^−2^ · s^−1^ photosynthetic photon flux density.

### RNA and genomic DNA isolation

Total RNA was extracted from different wheat tissues using Trizol reagent (Invitrogen, Carlsbad, CA) according to the manufacturer’s instructions. RNA concentration and purity were analyzed by Nanodrop ND-2000 spectrophotometer (Thermo Scientific, Wilmington, DE). The integrity of RNA sample was assessed by a non-denaturing agarose gel analysis. Genomic DNA was isolated from wheat leaves by cetyltrimethyl ammonium bromide (CTAB) extraction method [48].

### Cloning and bioinformatics analysis of *TaLCYB*

Total RNA extracted from wheat seedlings was used to synthesize cDNAs using RevertAid™ first-strand cDNA synthesis kit (Fermentas, Lithuania). In order to identify putative lycopene cyclase genes in wheat, a BLASTN search was performed with the sequences of *OsLCYB* from *O. sativa* (GenBank Accession No.: AP005849) [49], and *ZmLCYB* from *Z. mays* to identify the putative *LCYB* (GenBank Accession No.: AAO18661) [11]. A *Triticum aestivum* cDNA clone (WT009_F16) from cultivar Chinese Spring showed high identity with *OsLCYB* and *ZmLCYB*. Sequence analysis by ORF Finder showed that WT009_F16 contained the full-length ORF and amplified from wheat cDNA by PCR using specific primer pairs (Additional file 1: Table S2). Cycling parameters for RT-PCR were: 94°C for 3 min, 30 cycles of 94/58/72°C for 30/30/90 s, respectively, and 72°C for 10 min. Purified PCR products were cloned into pMD18-T simple vector (Takara, Dalian, China) then sequenced.

Prediction of transit peptide of TaLCYB was performed using ChloroP 1.1 Prediction Server program [50]. LCYBs sequences were searched at the NCBI (Bethesda, USA) and five amino acid sequences of LCYB were used for phylogenetic analysis. A phylogenetic tree was constructed by the Neighbor–Joining method [51] included in the ClustalW program [52] and bootstrap re-sampling analysis (1000 replicates) was performed.

### Functional characterization of wheat TaLCYB in *E. coli*

Full-length of the *TaLCYB* cDNA was cloned into pET-32a+. The plasmid pET-LCYB with pAC-LYC was used to transform *E. coli* strain. Plasmid pAC-LYC is a pACYC184 derived vector including several carotenoid biosynthesis genes, such as geranylgeranyl pyrophosphate synthase (*CrtE*), phytoene synthase (*CrtB*), and phytoene desaturase (*CrtI*) [8]. *E. coli* colonies containing pAC-LYC accumulate lycopene and appear pink. The co-transformants of plasmid pET-LCYB with pAC-LYC were plated onto LB agar medium added with chloramphenicol (50 μg ml^−1^) and ampicillin (100 μg ml^−1^). Colonies were incubated for 24 h at 37°C.

### Plasmid constructs

PCR primers were designed on the sequence of *TaLCYB* using the Primer 5. The pAHC25 containing the maize *ubi-1* promoter and the nopaline synthase terminator were used to construct RNAi vector. Fragments of 204 bp corresponding to *TaLCYB* were isolated by RT-PCR using specific primers with incorporated restriction sites (Additional file 1: Table S2). The selected fragments followed the selection strategies for RNAi in wheat [53]. Cycling parameters for PCR amplification were: 35 cycles of 94/62/72°C for 30/45/30 s, respectively, and 72°C for 10 min. The amplified fragments were then subcloned into pBluescript SK plus and sequenced. The fragments recovered by *Sma*I and *Not*I digestion were cloned in the same restriction site of plasmid pAHC25. RNAi construct contained a cDNA fragment derived from *TaLCYB* and oriented in the sense and antisense directions at the 3’ and 5’ ends of the construct separated by an intron sequence, respectively, and the resulting plasmid was named pAHC25-LCYB-RNAi (Additional file 1: Figure S5). The intron was derived from the wheat *TAK14* gene (AF325198).

### Wheat transformation and plant regeneration

Wheat genetic transformation was according to the bombardment method reported by Sparks [54]. Wheat immature scutella (14 DAP) from Chinese Spring were transformed with the plasmids of pAHC25-LCYB-RNAi or pAHC25 as a control. The regenerated plants were screened by the herbicide phosphinotricin medium (3 mg L^−1^). The surviving plants were transferred to soil and grown to maturity under growth chamber conditions (22°C/16°C day/night, 16/8-h light/dark cycle and 300 μmol · m^−2^ · s^−1^ photosynthetic photon flux density). The regenerated plants were continued to screen by PCR amplification using gene-specific primers (Additional file 1: Table S2). The PCR-positive transgenic plants were self pollinated and the non-segregant lines were selected to analyze the carotenoid profiles and expression levels of carotenoid biosynthetic genes (Additional file 1: Figure S6).

### qPCR analysis

The qPCR analysis was performed with the Realtime System (Bio-Rad, CFX Connect Optics Module, USA) using SuperReal PreMix Plus (SYBR Green) (FP205, Tiangen, Beijing, China). The amplification was performed with the following programme: 40 cycles of 95°C for 15 s, and 60°C for 60 s. Fluorescence was acquired at 60°C. The specificity of the unique amplification product was determined by a melt curve analysis from 55-99°C. Data were analyzed using the Lightcycler software version 4 and normalized to the expression of wheat β*-actin* gene as its relatively constitutive expression levels throughout wheat developmental process. The quality of the cDNA templates and PCR amplifications were verified by the analysis of negative controls without template and no-reverse transcription for each primer pair. Dissociation curve analysis was performed following qPCR and a single peak was observed for each primer pair. A portion of qPCR products was separated on agarose gels and single band at expected sizes were detected.

### Analysis of carotenoid composition by HPLC

Carotenoids in the mature wheat seeds were extracted according to Wang *et al.* [25] with some modifications. Seed samples were ground into fine powder. One gram powder was added with 15 mL extracting solvent (hexane: acetone: ethanol, 50:25:25, v/v/v) containing 0.01% (w/v) 2,6-di-tert-butyl-methylphenol (BHT, Sigma, Shanghai, China) following sonication for 30 min (SB-5200DTN, Scientz, China), and centrifuged for 10 min at 10,000 g under 4°C (CR-21G, Himac, Japan). The colored supernatant was collected, and the residue was re-extracted several times with extraction operation until colorless. The combined supernatant was then washed three times with saturated NaCl solution until neutral, and the aqueous phase was discarded. The solvent was evaporated under nitrogen stream, the pigments were redissolved in 0.3 mL methyltert-butyl ether (MTBE) containing 0.01% (w/v) BHT. After centrifuging at 12,000 rpm under 4°C for 30 min, the sample was filtered through a 0.22 μm filter before HPLC analysis. For quantitative purpose, β*-*apo-8’-carotenal was added to each sample as an internal standard prior to extraction (10 μg g^−1^ of freeze-dried sample). Carotenoids in the *E. coli* cells were extracted according to Alquezar [27].

The sample was injected into the HPLC system. The HPLC system included a model 2996 photodiode array detection (DAD) system, a 1525 solvent delivery system, and a Breeze2 Chromatography Manager (Waters Corpora-tion, Milford, MA. Carotenoids were separated by an YMC C30 carotenoid column (150 × 4.6 mm, packing 3 μm) (Wilmington, NC, USA) at 25°C. All the eluate was under 200 to 700 nm monitoring. The solvent A was acetonitrile/methanol (3:1, v/v), containing 0.01% BHT and 0.05% triethylamine (TEA, Sigma, Shanghai, China), and solvent B was 100% MTBE, containing 0.01% BHT. The parameters of mobile-phase gradient were programmed as follows: 0–10 min, A-B (95:5); 10–19 min, A-B (86:14); 19–29 min, A-B (75:25); 29–54 min, A-B (50:50); 54–66 min, A-B (26:74) and back to the initial condition for re-equilibration. All solvents were HPLC grade (J.T. Baker, Phillipsburg, USA). Carotenoid standards for lutein, zeaxanthin, β-cryptoxanthin, α-carotene, *trans*-β-carotene, β-apo-8’-carotenal, *trans*-lycopene calibration were purchased from Sigma-Aldrich (Shanghai, China); *9*-*cis*-β-carotene was purchased from Carotenature (Lupsingen, Switzerland). These standards, and the β-apo-8’-carotenal internal standard, were used to generate standard calibration curves. Carotenoids and chlorophylls were identified by comparing the retention time and spectra with published data and then quantified from their peak areas [55-58].

## Additional file

Additional file 1: Figure S1. HPLC characterization of carotenoids extracted from developing grains. **Figure S2.** Expression patterns of wheat *TaLCYB* in leaves with different treatments and β-carotene content varied with strong light. **Figure S3.** HPLC characterization of wheat leaf’s carotenoids composition under strong light treatment. **Figure S4.** Expression levels of the endogenous carotenoid biosynthetic genes in leaves with strong light treatment. **Figure S5.** The structures of transformation plasmids (pAHC25-LCYB-RNAi and pAHC25) used in this study. **Figure S6.** Propagation of transgenic wheat and selection of non-segregant lines of *TaLCYB* silencing. **Table S1.** Cartenoids content and compositions in T_2_ seeds from the transgenic and control wheat plants. **Table S2**. Primer sequences used in this study.

## Abbreviations

BHT: 2,6-di-tert-butyl-methylphenol
β-LCY CAD region: β-LCY Catalytic Activity Domain region
CCDs: Carotenoid cleavage dioxygenases
CrtB: Phytoene synthase
CrtE: Geranylgeranyl pyrophosphate synthase
CrtI: Phytoene desaturase
CTAB: Cetyltrimethyl ammonium bromide
DAD: Photodiode array detection
DAP: Days after pollination
HPLC: High Performance Liquid Chromatography
HYD: β-carotene hydroxylase
LCYB: Lycopene β-cyclase
LCYE: Lycopene ε-cyclase
MTBE: Methyl tert-butyl ether
PDS: Phytoene desaturase
PSY: Phytoene synthase
qPCR: Quantitative PCR
RNAi: RNA interference
TEA: Triethylamine
VAD: Vitamin A deficiency
VC: Vector control
ZDS: Zeta-carotene desaturase.
