# Supplementary material for: The lycopene β-cyclase plays a significant role in provitamin A biosynthesis in wheat endosperm
Source: BMC Plant Biol. 2015 May 7;15:112. doi: 10.1186/s12870-015-0514-5 (PMC4433027; doi:10.1186/s12870-015-0514-5)
Supplement: Additional file 1: Figure S1. — HPLC characterization of carotenoids extracted from developing grains. Figure S2. Expression patterns of wheat TaLCYB in leaves with different treatments and β-carotene content varied with strong light. Figure S3. HPLC characterization of wheat leaf’s carotenoids composition under strong light treatment. Figure S4. Expression levels of the endogenous carotenoid biosynthetic genes in leaves with strong light treatment. Figure S5. The structures of transformation plasmids (pAHC25-LCYB-RNAi and pAHC25) used in this study. Figure S6. Propagation of transgenic wheat and selection of non-segregant lines of TaLCYB silencing. Table S1. Cartenoids content and compositions in T2 seeds from the transgenic and control wheat plants. Table S2. Primer sequences used in this study. [file 12870_2015_514_MOESM1_ESM.pdf]

Additional file

Figure S1. HPLC characterization of carotenoids extracted from developing grains. (A)-(E): Grain 1 (4–10 DAP), grain 2 (10–16 DAP), grain 3 (16–20 DAP), grain 4 (20–25 DAP) and grain 5 (25–35 DAP); Peak 1, Chlorophyll b; Peak 2, lutein; Peak 3, Zeaxanthin; Peak 4, Chlorophyll a; Peak 5, *β*-crptoxanthin; Peak 6, *α*-carotene; Peak 7, *trans*-*β*-carotene; Peak 8, 9-*cis*-*β*-carotene;

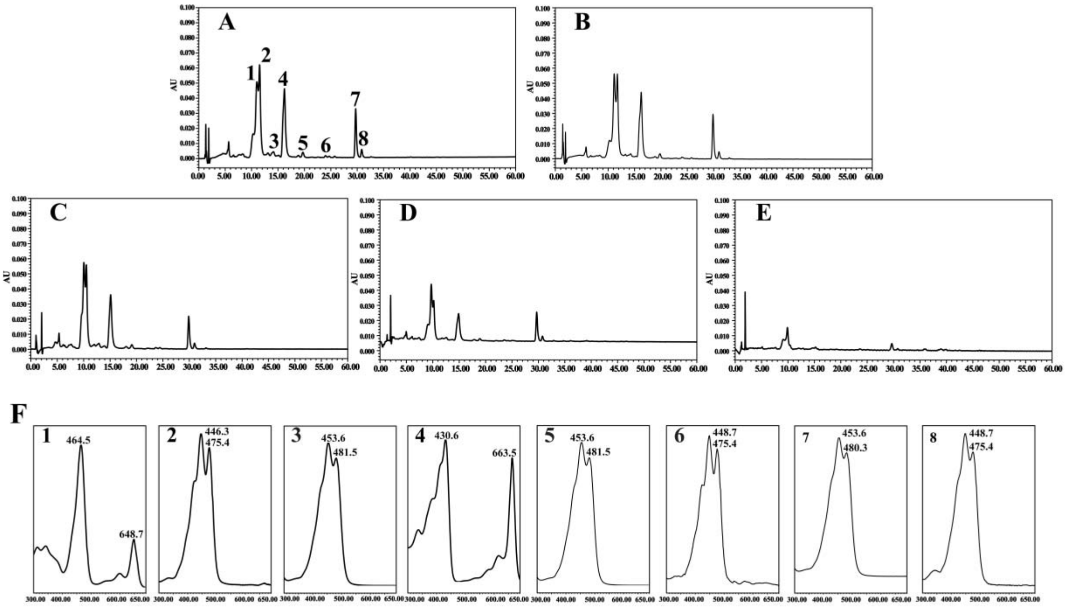

Figure S2. Expression patterns of wheat *TaLCYB* in leaves with different treatments and β-carotene content varied with strong light. (A) The seedlings were exposed to various physiological stresses including strong light, darkness and cold. Poly(A)$^+$ mRNA of 200 ng was subjected to reverse transcription, and served as the qPCR template. qPCR results for each gene were performed in three biological replicates with three technical repeats each and all data are shown as Mean ± SEM. (B) β-carotene content changed with various hours of strong light. Single asterisk indicates significant differences in the β-carotene content levels between controls and treatment at P = 0.05 probability level.

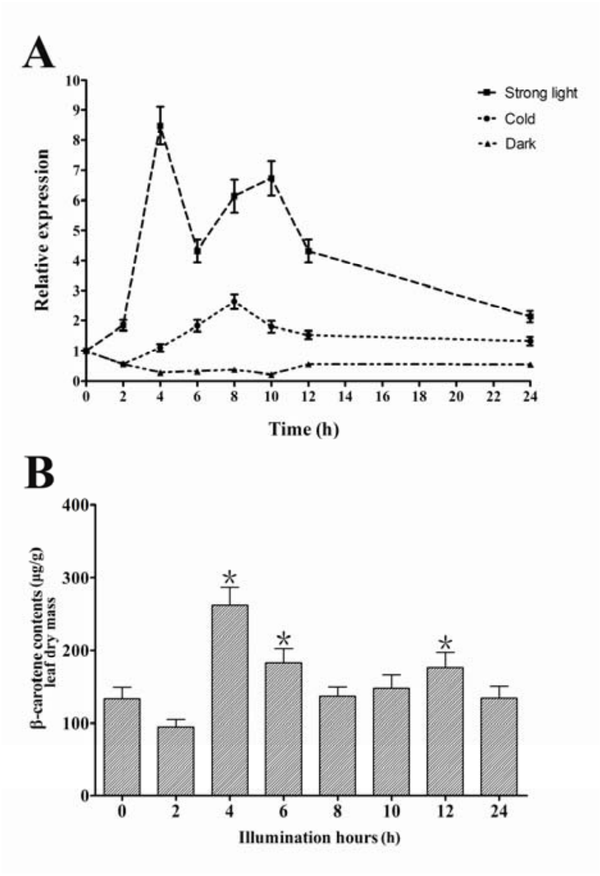

Figure S3. HPLC characterization of wheat leaf's carotenoids composition under strong light treatment. (A)-(H): 0 h; 2 h, 4 h, 6 h, 8 h, 10 h, 12 h, 24 h; Peak 1, Chlorophyll b; Peak 2, lutein; Peak 3, Chlorophyll a; Peak 4, *trans-β*-carotene; Peak 5, 9-*cis-β*-carotene;

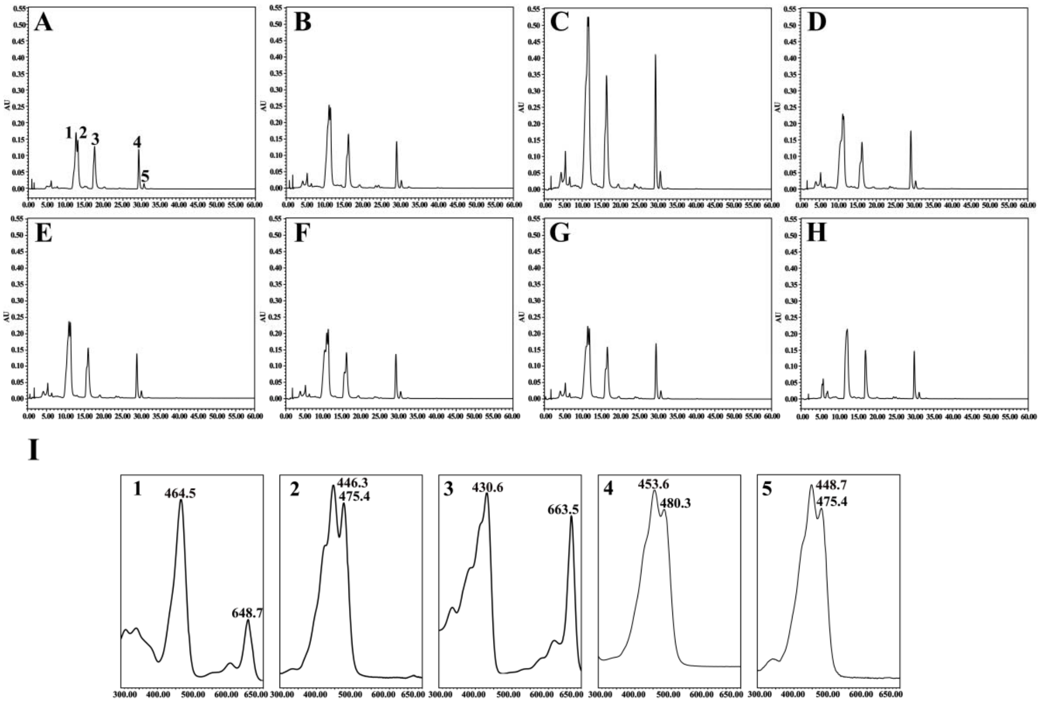

Figure S4. Expression levels of the endogenous carotenoid biosynthetic genes in leaves with strong light treatment.

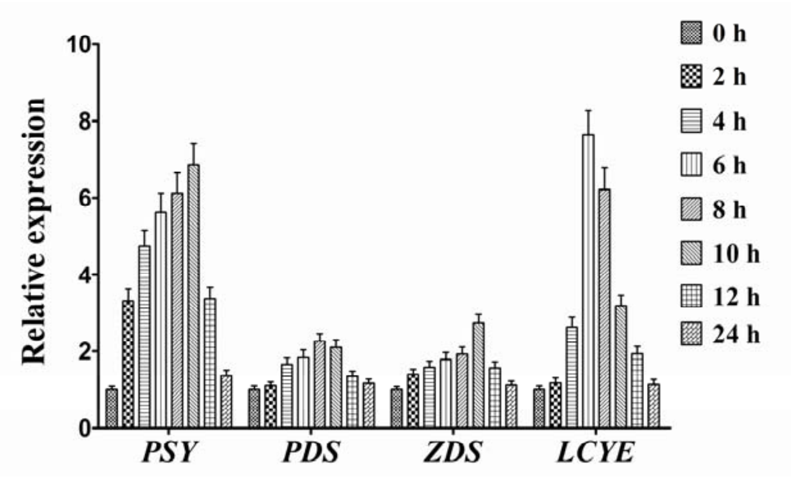

Figure S5. The structures of transformation plasmids (pAHC25-LCYB-RNAi and pAHC25) used in this study. *Ubi*, constitutive maize *Ubiquitin-1* promoter, *NOS*, *Agrobacterium tumefaciens* nopaline synthase (*NOS*) terminator. Sense and antisense, fragment of *TaLcyb*, designed to silence the expression of the *TaLCYB* gene; Intron, fragment of *TAK1*4, *Bar*, Bialaphos resistance gene.

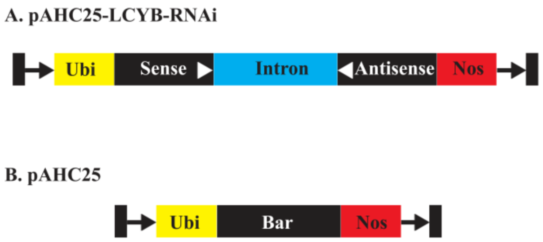

Figure S6. Propagation of transgenic wheat and selection of non-segregant lines of *TaLCYB* silencing. Selection of transgenic plants in T_0 generation by PCR led to three T_0 plants (designated BI-2, BI-6 and BI-9 plants). Seeds from each single plant were harvested separately in the following generations. Because very few seeds were harvested from each T_0 plants, one or two T_1 seeds from each T_0 plant were planted to generate T_2 seeds. To evaluate the changes in carotenoid compositions in transgenic wheat, the T_2 seeds from the top-three T_1 plants that produced most seeds were used for analysis of the carotenoid contents and compositions by HPLC, with eight seeds from each of these T_1 plants being planted to produce T_2 plants. Then selection of non-segregant lines for transgens by PCR in T_1 and T_2 generations revealed three transgenic lines, BI-2, BI-6 and BI-9. Their seeds and leaves in T_3 generation were used for analyzing carotenoid compositions by HPLC and quantifying expression levels of carotenoid biosynthetic genes by qPCR.

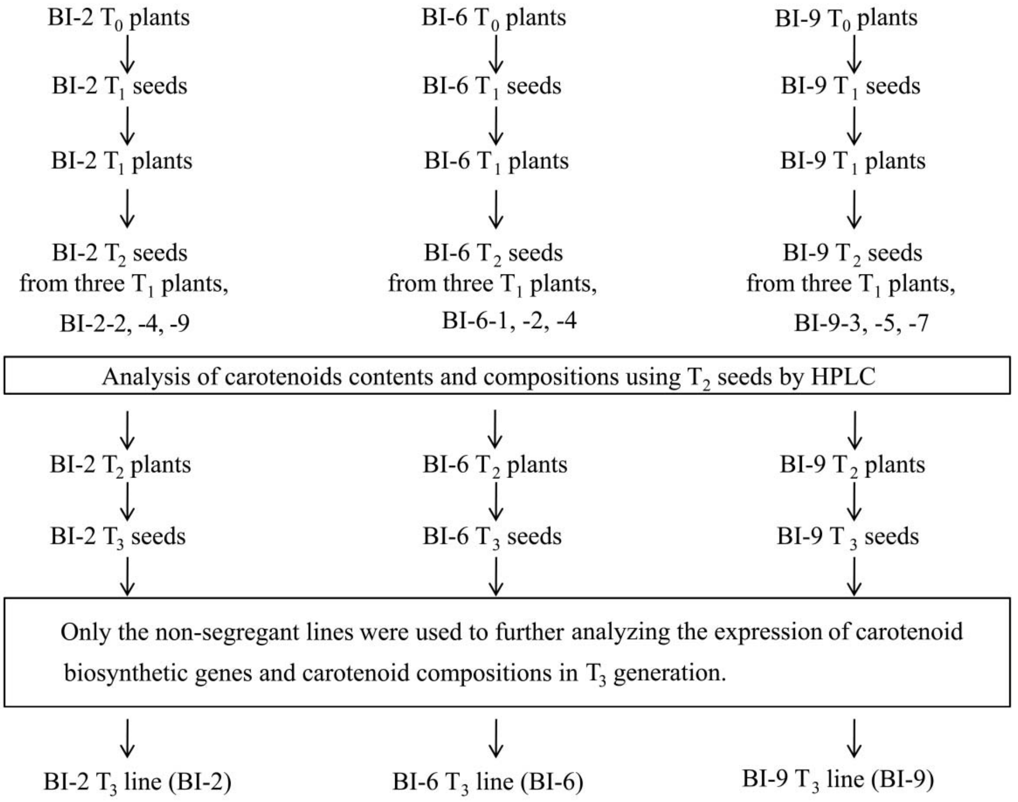

Table S1. Cartenoids content and compositions in T_2 seeds from the transgenic and control wheat plants[a]

Lines [b] (T_2)	Lutein ($\mu g\ g^{-1}$)	Zeaxanthin ($\mu g\ g^{-1}$)	β-cryptoxanthin ($\mu g\ g^{-1}$)	Lycopene ($\mu g\ g^{-1}$)	α-carotene ($\mu g\ g^{-1}$)	β-carotene ($\mu g\ g^{-1}$)	Phytoene ($\mu g\ g^{-1}$)	Total ($\mu g\ g^{-1}$)
Chinesespring	0.58 (59%)[c]	0.16 (16%)	ND	ND	ND	0.24 (25%)	ND	0.98
VC-8	0.56 (59%)	0.17 (18%)	ND	ND	ND	0.22 (23%)	ND	0.95
BI-2-2	0.52 (55%)	0.17 (18%)	ND	ND	ND	0.26 (27%)	ND	0.95
BI-2-4	0.55 (56%)	0.15 (15%)	ND	ND	ND	0.28 (29%)	ND	0.98
BI-2-9	0.54 (56%)	0.17 (18%)	ND	ND	ND	0.25 (26%)	ND	0.96
BI-6-1	0.23 (26%)	0.15 (17%)	0.05 (6%)	0.21 (24%)	0.06 (7%)	0.17 (20%)	ND	0.87
BI-6-2	0.20 (24%)	0.13 (15%)	0.06 (7%)	0.22 (26%)	0.07 (8%)	0.16 (19%)	ND	0.84
BI-6-4	0.22 (26%)	0.16 (19%)	0.07 (8%)	0.20 (23%)	0.06 (7%)	0.15 (17%)	ND	0.86
BI-9-3	0.19(24%)	0.06(8%)	ND	0.38 (48%)	0.06 (7%)	0.10(13%)	ND	0.79
BI-9-5	0.20 (25%)	0.05 (6%)	ND	0.39 (49%)	0.04 (5%)	0.11 (14%)	ND	0.79
BI-9-7	0.19 (24%)	0.07 (9%)	0.02 (3%)	0.36 (45%)	0.07 (8%)	0.09 (11%)	ND	0.80

[a] Due to seed limitation in T_2 generation, carotenoid content and composition for the T_2 transgenic lines and the wild-type were determined by HPLC without replication, primarily estimating the effects of down-regulating expression of *TaLCYB* on carotenoid accumulation in wheat kernels;

[b] Chinese Spring is the untransformed wheat cultivar (wild-type); VC-8 is a wheat line transformed with plasmid pAHC25, which is used as transgenic control line; All the lines with labeled BI are T_2 lines with expression of interference vector from BI-2, BI-6 and BI-9;

[c] Values in parentheses represent the percentages of each carotenoid composition relative to the total content;

ND= not detected.

Table S2. Primer sequences used in this study

Primers name	Gene accession No.	Primer sequences (5'-to-3' direction)	Application
TaLCYB-F	JN622196	ATGGCCACCACCGCCCT	*TaLCYB* ORF
TaLCYB-R	JN622196	TCACCTATCCTTGTCCTGTATCA	*TaLCYB* ORF
*Sma*I-LCYB-S-F	JN622196	TCCCCCGGG CGCCGAGGTGTGGAAGGAG	Vector construction of RNAi
*Bam*HI-LCYB-S-R	JN622196	CGGGATCC GAGAGCCCAAACATCAAGAGC	Vector construction of RNAi
*Spe*I-LCYB-AS-F	JN622196	GGACTAGT GAGAGCCCAAACATCAAGAGC	Vector construction of RNAi
*Not*I-LCYB-AS-R	JN622196	TTGCGGCCGC CGCCGAGGTGTGGAAGGAG	Vector construction of RNAi
*Spe*I-Intron-F	AF325198	CGGGATCC TTGTGCCAGATATTTGCTCC	Vector construction of RNAi
*Not*I-Intron-R	AF325198	GGACTAGT TGATATCCGTTCTGTTTCTA	Vector construction of RNAi
Bar-F	X05822.1	GTCTGCACCATCGTCAACC	PCR confirmation of wheat transformants
Bar-R	X05822.1	GAAGTCCAGCTGCCAGAAAC	PCR confirmation of wheat transformants
Actin-F	AB181991	AGTGGAGGTTCTACCATGTTTCCT	Quantitative PCR analysis
Actin-R	AB181991	CACTGTATTTCCTTTCAGGTGGTG	Quantitative PCR analysis
PSY-F	EF600063	GTTTGGGCCTCTCTGTTGTTG	Quantitative PCR analysis
PSY-R	EF600063	GCCCTCTTGGTGAAGTTGTTG	Quantitative PCR analysis
PDS-F	FJ517553	TGAACGCCCCAGTAAACCA	Quantitative PCR analysis
PDS-R	FJ517553	TTTCCGCCCAACACATCTC	Quantitative PCR analysis
ZDS-F	FJ169496	TTTAGACCTGACCAGAAGACACCA	Quantitative PCR analysis
ZDS-R	FJ169496	AATAACTCCTCTCCAGCACCACA	Quantitative PCR analysis
LCYB-F	JN622196	CGACGGTTCTTCAACGCATTCTT	Quantitative PCR analysis
LCYB-R	JN622196	TCCTGTATCAAGTTGCCGACCAT	Quantitative PCR analysis
LCYE-F	EU649786	ACACACCCTGAGGAAGCCAA	Quantitative PCR analysis
LCYE-R	EU649786	CGCATCCAACCGAGACATCAAC	Quantitative PCR analysis
CrtI-F	D90087	CGAAGGTTATCGTCAGTTTCTGG	Quantitative PCR analysis
CrtI-R	D90087	GCAGTTTCGCCAGTTGAGG	Quantitative PCR analysis

CrtB-F	D90087	CTGATGCTCTACGCCTGGTG	Quantitative PCR analysis
CrtB-R	D90087	CGTTGTTCGGGCGTTTG	Quantitative PCR analysis
HYD1-F	JX171671	ACCACATGGACAAGTTCGAGG	Quantitative PCR analysis
HYD1-R	JX171671	TTGATCCTGGCGAGCTCCT	Quantitative PCR analysis
HYD2-F	JX171673	AAGTTCGACAGCGTGCCATAC	Quantitative PCR analysis
HYD2-R	JX171673	TCCTGATCCTCCTCTGCACCT	Quantitative PCR analysis
